# Excess Mortality Due to External Causes in Women in the South African Mining Industry: 2013–2015

**DOI:** 10.3390/ijerph17061875

**Published:** 2020-03-13

**Authors:** Kerry S. Wilson, Tahira Kootbodien, Nisha Naicker

**Affiliations:** 1 Epidemiology and Surveillance Section, National Institute for Occupational Health, 25 Hospital St, Constitution Hill, Johannesburg 2000, South Africa; tahirak@nioh.ac.za (T.K.); NishaN@nioh.ac.za (N.N.); 2 School of Public Health, Faculty of Health Sciences, University of the Witwatersrand, 9 York Rd, Parktown, Johannesburg 2193, South Africa; 3 Department of Environmental Health, Faculty of Health Sciences, University of Johannesburg, PO Box 524, Auckland Park 2006, South Africa

**Keywords:** women miners, mortality, external causes of death, age, proportional mortality ratios

## Abstract

Mining is a recognized high-risk industry with a relatively high occurrence of occupational injuries and disease. In this study, we looked at the differences in mortality between male and female miners in South Africa. Data from Statistics South Africa regarding occupation and cause of death in the combined years 2013–2015 were analyzed. Proportional mortality ratios (PMRs) were calculated to investigate excess mortality due to external causes of death by sex in miners and in manufacturing laborers. Results: Women miners died at a significantly younger age on average (44 years) than all women (60 years), women manufacturers (53 years), and male miners (55 yrs). There was a significantly increased proportion of deaths due to external causes in women miners (12.4%) compared to all women (4.8%) and women manufacturers (4.6%). Significantly increased PMRs were seen in car occupant accidents (467, 95% confidence interval (CI) 151–1447), firearm discharge (464, 95% CI 220–974), and contact with blunt objects (2220 95% CI 833–5915). Conclusion: This descriptive study showed excess deaths in women miners due to external causes. Road accidents, firearm discharge, and contact with blunt objects PMRs were significantly increased. Further research is required to confirm the underlying reasons for external causes of death and to develop recommendations to protect women miners.

## 1. Introduction

In 2002, the Mineral and Petroleum Resources Development Act (MPRDA) of 2002 and the Mine Health and Safety Act of 1996 opened mines in South Africa to women [1]. Following this, the 2004 South African Mining Charter recognised that in South Africa, women were historically prevented from participating in the mainstream economy. Thus, plans were put in place to “ensure higher levels of inclusiveness and advancement of women” [2]. In 2017, the Minerals Council South Africa Women in Mining report showed that 12% of miners were women, with 14.9% in top management [3]. 

Mining globally and in South Africa is considered a hazardous industry with a number of risks, from exposure to crystalline silica to accidents and ground movements [4,5]. In China, increased all-cause and cause-specific mortality of miners was found in a large follow-up cohort study of silica-exposed and silica-non-exposed workers [6]. Further, in the USA, fatal injury rates in mining were found to be four times higher than the average for all industries [7]. In South Africa, studies reported miners to be at increased risk of Tuberculosis (TB) and Human Immunodeficiency Virus (HIV) [8,9], respiratory diseases [10,11,12,13], and injuries [14,15,16]. In a 2008 mortality cohort study of platinum miners in South Africa, external causes were found to be the second most common cause of death after HIV [15]. Road traffic accidents were the most common cause of unnatural death in the study (38%), followed by homicide (30%) and occupational injuries (17%). Despite this finding, men in the cohort exhibited lower rates of unnatural deaths overall than the average South African population (Incidence rate ratio 0.89, 95% confidence interval (CI) 0.82–0.95) [15]. A recent study by Bloch et al. (2018) found that women miners did not have excess mortality compared to male miners. They reported an excess of mortality overall in miners which was 20% higher than the general public, although they found that the mortality rate decreased over time [16]. Although research on mortality in mining generally focuses on men, recent reports by Benya et al., 2019 highlighted the violence faced by women due to a masculine occupational culture in mining [17,18]. 

Thus, with the dangers associated with working in the mining industry and the additional vulnerability of women miners, we aimed to determine the extent (prevalence) and cause of mortality in women miners in South Africa using the national death registry data.

## 2. Materials and Methods

Mortality, usual occupation, and industry data, as captured from death certificates in South Africa from 2013 to 2015, were retrieved from the website for Statistics South Africa (www.statssa.gov.za). The data, consisting of deaths due to underlying cause, which was coded by Stats SA using the 10th International Classification of Diseases Codes, were used to calculate the proportions of death according to the main cause of death for each occupational group. The occupation was coded by Statistics South Africa using the South African Standard Classification of Occupations (SASCO) list [19]. The information for this field was obtained from the open question asked on page one of the DHA-1663A form, part A, question 19, i.e., “What was the usual occupation of the deceased (the type of work done during most of life)?” [20]. This analysis was limited to three years (2013–2015), as the occupation was not coded in detail from 1997 to 2013, therefore the miners could not be identified. Detailed coding for occupation in 2016 mortality data is not currently available. These data are publicly available and an ethics waiver was received from the University of the Witwatersrand Human Ethics Committee for the secondary analysis of these data. 

Occupations included the group miners, including mining supervisors, mining and mineral processing plant operators, metal processing operators, stationary plant operators, machinery mechanics, electrical installers (within the mining industry), building finishers (within the mining industry), mining laborers, and other elementary workers (within the mining industry). For occupational groups not specific to the mining industry, only those who also reported mining as the industry they worked in were included. The largest occupation reported in the mining industry for this dataset was mining and mineral processing (61%). 

Male and female manufacturing laborers were chosen as a comparison group for miners as they were classified in the same occupation subgroup (construction, manufacturing, and mining are grouped together in an occupational subgroup in SASCO). These workers also often undertake manual labor and are exposed to chemical hazards in their work, although these hazards are of a different nature. Manufacturing workers are likely to be of similar social and socio-economic status in South Africa, thus, they may exhibit differences due to their occupations. For comparison, men and women who were reported to be unemployed were included, while those who presented no information regarding occupation were excluded.

### 2.1. Data Management

**Figure 1:**
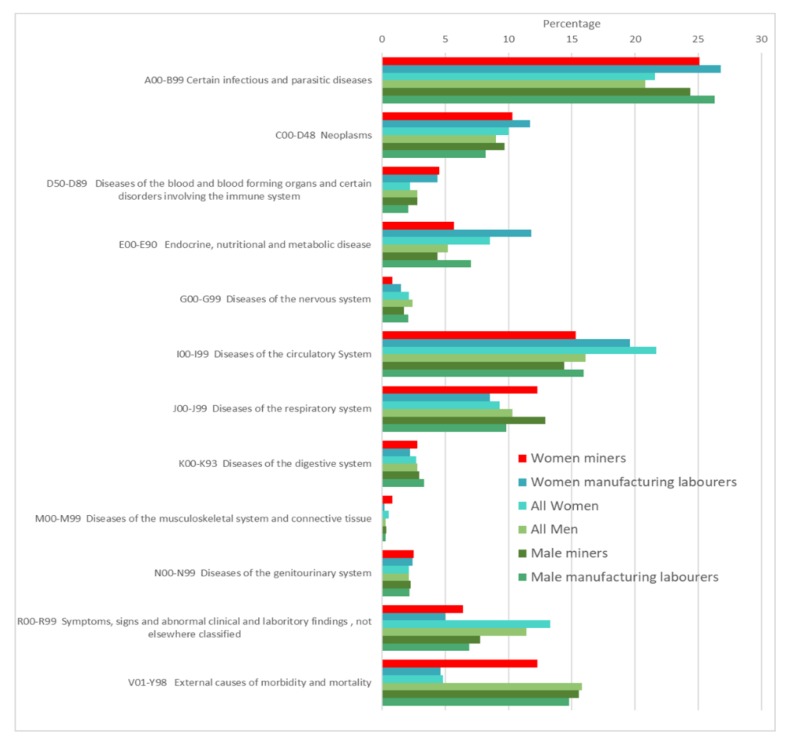
Percentages of deaths in the main groups (ICD 10) of underlying causes of death in South Africans aged 15+ for women miners, women manufacturing laborers, all women, all men, male miners and male manufacturing laborers over the combined years 2013–2015.

The South African mortality data from the years 2013–2015 were combined to investigate the mortality experience of women miners due to the small numbers of women working in the mines as miners. The variables used in the analysis were cleaned by recoding any unknown values in age, marital status, education, or sex to “missing” so that the numbers used to code this information were not included in calculations. Deaths of persons aged 14 and below were removed from the dataset, as this analysis focused on the possible underlying causes of death linked to occupation or exposure from work. In South Africa, 15 years of age is the legal working age. This study was limited to those who died 15 years and older. A cut-off of 65 years (retirement age) was not used to account for causes of death with long lag phases, such as cancer and pneumoconiosis. Marital status was condensed into ever married or never married. Education was summarized into four groups, namely, none, primary school, high school, or tertiary education (Table 1). Both the minor occupation (coded to three digits) and the industry were used to identify mining industry workers, manufacturing laborers, and those who were unemployed. The proportion by occupation for underlying group causes (ICD10) with proportions above 1% for ages 15 and above are presented in Figure 1. The external causes of death analyses in Table 2 include all external causes of death reported in women miners and the total external causes of death, which also corresponded to the most common external causes of death in the entire dataset. Mortality in section P00-P96 was not reported, as this was specific to the perinatal period.

### 2.2. Statistical Analysis

Data analysis was conducted using STATA software v16 (Stata Statistical Software: Release 16 SE, StataCorp. 2017. StataCorp LLC, College Station, TX, USA) and Microsoft Excel 2010. Student’s *t*-tests were used to compare means, while Wilcoxson rank sum (RS) was used to compare medians and proportion tests were used to compare proportions within the groups. The alpha level was set at 0.05. 

Proportional mortality ratios (PMRs) were calculated for the external causes of death for women miners, women manufacturing labourers, male miners, and male manufacturers using total deaths in all reported working women and all reported working men as denominators. The number of current workers in each of these categories is not currently available; thus, it was not possible to calculate mortality rates. The 95% confidence intervals (95% CI) were calculated for each estimate using the method for calculating 95% CIs for rates. 

Proportional mortality (PM):

(1) $$\frac{Number\;of\;deaths\;due\;to\;a\;specific\;cause\;in\;a\;population}{Total\;number\;of\;deaths\;from\;all\;causes\;in\;the\;same\;population\;}$$

Proportionate mortality ratio (PMR):

(2) $$\frac{Proportionate\;mortality\;for\;a\;specific\;cause\;in\;the\;population\;of\;interest}{Proportional\;mortality\;for\;the\;same\;cause\;in\;the\;general\;population}\;\times \;100$$

A PMR above 100 was considered increased and significant if the 95% CI did not include 100. 

A multivariate logistic regression was performed for sensitivity analysis, using backward regression with the available variables. Variables were retained in the model if they were significant.

## 3. Results

There were approximately 1,247,000 registered deaths from 2013 to 2015. A total of 8362 (0.69%) deaths in miners were identified. The demographic characteristics for miners and manufacturing laborers were compared to those of all men and women of working age (Table 1). 

Generally, women died at a significantly older average age than men 9 (difference of eight yrs, Wilcoxon RS *p* < 0.001). In contrast, female miners died younger than men (- 9 yrs, Wilcoxon RS *p* < 0.0001), male miners (- 11 years, Wilcoxon RS *p* < 0.0001), female manufacturing laborers (- 9 yrs, *t*-test *p* < 0.0001) and male manufacturing laborers (- 8 years, Wilcoxon RS *p* < 0.0001). Women miners died sixteen years younger on average than all women (*p* < 0.0001), while male miners died at an older age than men in general (1.4 years, Wilcoxon RS *p* < 0.0149) and were older than manufacturing laborers. 

Women miners were more educated than women in general (proportions test, *p* < 0.0001), and achieved a higher level of education than male miners (proportions test *p* < 0.0001). Women miners were generally less likely to be married than men and significantly less likely than male miners (proportions test *p* < 0.0001). There was no significant difference in smoking history between women miners and women in general (proportions test *p* = 0.3044); however, women miners were less likely to smoke than men in general and male miners (proportions test *p* < 0.0001). 

### Underlying Causes of Death

All causes of death were investigated, i.e., not only occupational deaths, as occupation may have an impact on health over and above occupational exposures. The medium quality of the data also limited any specific analyses [21]. Investigating the underlying causes of death in female miners is complicated, as women in South Africa generally exhibit different mortality patterns to men and miners display different patterns to non-miners. Thus, Figure 1 compares the proportions of deaths in both women and male miners and non-miners.

Women miners and women manufacturing laborers showed different patterns regarding causes of death compared to women in general. Working women displayed increased proportions of deaths due to infectious diseases, with a 3.5% increase in women miners and 5.2% in women manufacturing laborers. Male miners and male manufacturing laborers also showed increases in infectious diseases deaths compared to all men. Male miners showed a 3.6% increase, which was similar to women miners, and male manufacturing laborers showed an increase of 5.5%, which was similar to women manufacturing laborers. Both groups of employed men and women exhibited reduced proportions of ill-defined deaths. Patterns showed a reduction of 6.4% in women miners and 8.3% in women manufacturing laborers, while male miners and male manufacturing laborers were 3.7% and 4.9% less likely to die of ill-defined causes, respectively. Following this pattern, women and male miners showed decreased proportions of deaths (6.4% and 1.7% reductions, respectively) due to diseases of the circulatory system compared to all men and women, while male and female manufacturing laborers exhibited smaller decreases (0.2% and 2.1% respectively). 

Contrary to the diseases above, women and male miners both suffered increased deaths due to diseases of the respiratory system (3% and 2.6% increases, respectively). However, male and female manufacturing laborers showed slight decreases in respiratory disease deaths (0.5% and 0.8%, respectively). 

Women miners showed an increased proportion of deaths due to external causes compared to all women (7.6% increase) and to women manufacturing laborers (7.7% increase), while male miners and male manufacturing laborers demonstrated similar proportions to all men. We reported all external causes of death, not only those reported as occupational incidents. 

External causes of death in this dataset counted for 10.5% of overall deaths, with men accounting for 78% of these deaths. Women miners suffered more than double the percentage of deaths (12.4%) due to external causes compared to all women (4.75%) and women manufacturing laborers (4.6%). The specific external causes of death were investigated further and are presented in Table 2.

We calculated proportional mortality ratios (PMRs) for all working women as the comparison group against women miners, women manufacturing laborers and unemployed women. All working men were the comparison group for the PMR calculations of male miners, male manufacturing laborers, and unemployed men. The PMRs indicated whether the proportion of deaths due to a specific external cause were high or low for the particular industry compared to all industries. 

Women miners presented a significantly increased PMR of 183 compared to all working women regarding total external causes of death. In contrast to this, the comparison groups of male miners, women, male manufacturing laborers, and unemployed women exhibited significantly decreased PMRs for external causes of death; only unemployed men showed a significantly increased PMR of 152.

The underlying causes were further investigated by focusing in detail on the causes reported in women miners. Women and male miners showed significantly increased PMRs for transport accidents, with the largest PMR of 467 reported for women miner car occupant accidents and a PMR of 165 for male miners. These deaths showed nonsignificant or decreased PMRs in the comparison groups. Women miners also exhibited a significantly increased PMR 243 for unspecified vehicle accidents, while unemployed men and women were protected and manufacturing laborers similar to employed workers in this regard. 

Unexpectedly, women miners had a significantly increased PMR of 464 for deaths due to firearm discharge. Firearm deaths in male miners were nonsignificantly reduced with a PMR 85, and nonsignificantly reduced in both women and male manufacturing laborers. The only other group with an increased PMR (186) for firearm deaths was unemployed men. The PMR for exposure to unspecified forces was significantly increased in women miners with a PMR 218, while reductions were observed in male miners, unemployed females, and male and female manufacturing workers. Again, only unemployed males showed a significant increase in PMR, which was 122. 

For deaths due to contact with a blunt object, women miners exhibited a significantly increased PMR of 2220, similar to that of unemployed women who showed a PMR of 1180, while women manufacturing laborers, male miners, and male manufacturing laborers did not exhibit significant increases. 

In the sensitivity analysis, which is presented in Table 3, there is an increase in the odds ratio of women miners compared to male miners after adjusting for age, marital status, and education, although there was poor model fit. 

## 4. Discussion

This study provided a profile of causes of death among women miners in South Africa with a focus on external causes of death. We reported excess unnatural deaths in women miners from transport accidents, firearm discharge, and contact with a blunt object at younger ages than both men and women in general. 

Women only recently were able to be employed as miners in the South African mining industry. It was and still is thought that mining is not a woman’s job [17], although by 2017 at least 12% of miners were female. In a study by Bloch et al. (2018) using mining recruiter data, ex-miner mortality from 2001–2013 was investigated, where 5.1% of ex-miners were female [16]. In our study of registered deaths from 2013 to 2015, a similar proportion (5.7%) of miners were female. Bloch et al. (2018) found no excess mortality in women ex-miners, while an average of 20% excess mortality was found in men. Their finding of a lack of excess mortality in women miners was limited by the fact that the mortality rate in ex-miners decreased over the years in the study and women were only employed in the later years. Also, as women miners generally were not exposed to silica for the same length of time as male ex-miners, they were less likely to exhibit increased deaths due to respiratory disease. Bloch and colleagues (2011) did find greater excess mortality amongst the youngest miners; it was further suggested, based on the findings of Lim et al. (2011), that violence and accidents play an important role in the deaths in this group [15]. 

It is commonly accepted that women generally live longer than men in developed and many developing countries [22,23]. Some studies demonstrated that this difference was likely to be mediated by social, behavioural, and environmental factors [22,24]. Contrary to these reports, this study found that South African women miners died at a significantly younger age on average than all South African working women, women manufacturing laborers, all working men, and male miners, while all women and all working women died at older ages than all men and all working men, following the expected trend. Tobacco consumption was identified as the most contributary factor behind the sex differences in mortality observed in many countries [24]. However, men’s participation in risky jobs was also recognised. Recently, these behaviours have been changing, as more women smoke and participate in work in risky industries, which is expected to reduce the mortality difference between men and women [23]. Despite more women taking up smoking, this behavior is unlikely to explain the reduction in longevity seen here in women miners, as the proportion of women miners who smoked was similar to that of all women and all working women and significantly lower than men. Our finding of a “reduction in median age at death” to 44 years in women miners was similar to the findings of a recent autopsy study by Kgokong et al. (2018), who reported that South African female miner’s mean ages at death were 37.8 and 39 years in the gold and platinum industries, respectively. They also reported a high incidence of unnatural deaths, which was similar to our findings [25]. The Pathaut database (hosted by the National Institute for Occupational Health) which collects autopsy results for South African miners whose organs are sent for autopsy, is biased toward miners who die during employment; despite this, the findings of this analysis regarding the national women miner mortality data support the findings of the Pathaut database analysis.

In this national dataset, women miners suffered from a high proportion of deaths due to infectious disease compared to all women and all men. This could be linked to the increased prevalence of HIV and TB in the mining industry or better access to health care, as seen in the reduction of ill-defined natural causes of death [9,26,27]. Women miners also suffered an increased proportion of deaths due to respiratory disease compared to all working women and women manufacturing laborers, while male miners showed a similar increase in respiratory disease deaths. This association between mining and respiratory disease is well described in a range of national and international literature. [13,26,27,28,29]. Women and male miners appear to be protected from endocrine and metabolic diseases and circulatory system deaths compared to women and men manufacturing laborers and all women and all men. This may be partly due to the physical nature of the work [30,31]. Finally, women miners exhibited a substantially significant increase in the proportion of deaths due to external causes compared to all women, working women, and women manufacturing laborers, while male miners showed a similar proportion to all men and male manufacturing laborers. 

Injury-related mortality in South Africa accounted for 12% of deaths and 16% of years of life lost in the first Burden of Disease Study in 2000 [32], primarily due to high mortality rates from road traffic injuries and homicides, which were approximately twice and eight times higher than global figures, respectively [33,34]. The second Burden of Disease Study reported that 9.6% of deaths were due to injuries, which was similar to the 10.53% of deaths classified as external causes of mortality in our data [35]. In 2015, Matzopoulos et al. reported elevated age-standardised mortality based on forensic post-mortem investigations, specifically, 38.4 years for homicide (95% CI: 33.8–43.0) and 36.1 years for road traffic injury (95% CI: 30.9–41.3) in South Africans, which were similar to our findings for men and women miners [34]. Despite the reduction in deaths due to mining accidents seen in recent years in developed countries and in South Africa [15,36], mining presents one of the highest rates of fatal occupational accidents among the industrial sectors [37]. 

Women miners exhibited increased PMRs for most types of vehicle-related accidents, firearm discharge deaths, and contact with a blunt object compared to women and men manufacturing laborers, male miners, and unemployed women. Transportation accidents were previously found to be a leading cause of occupational fatalities in the mining sector compared to all other industries, and often in younger workers. Janicak et al. (2011) suggested that younger workers may have less experience and/or may be assigned more hazardous jobs [38]. This combined with the risks inherent in traveling long distances as migrant workers visiting home for weekends and holidays may explain this increase [15] and may contribute to the increased numbers of younger women miners appearing in our dataset. The recent report from Action Aid 2019 described an increase in gender-based violence in mining communities as the industry attracts large numbers of migrant men as workers, which can force some women into transactional sex to survive and contribute to an unsettled community. The report identified substance abuse as one factor in mining community violence [39]. The recent review by Botha (2016) on the continued harassment and exploitation of women miners supports the validity of the increased PMRs for firearm discharge and contact with a blunt object in women miners in our study, along with the findings of the Action Aid report [40]. 

Women miners were less likely to die from unspecified natural causes, but more likely to die from unspecified external causes. Exposure to unspecified forces is the same as ill-defined regarding disease, and should only be used when there is absolutely no information on the cause of death. Literature reported that women are at increased risk of sexual harassment and violence underground and women are also often exposed to equipment not designed for them [3,18,40]

Vehicle accidents were the only external cause of death where male miners exhibited a significantly increased PMR, which corresponds to the findings of Lim et al. 2011 who determined a high mortality rate for their cohort of male miners in a platinum mine due to unnatural causes relative to the world average, but a lower rate than South African men in general. Lim et al. also reported that road traffic accidents were the most common cause of unnatural death in the mining cohort.

### Strengths and Limitations of This Study

The South African death registry provides coverage of the entire country, with approximately 93% of all deaths registered [41]. All death records with industry and occupation data were included in the study, therefore, the limitations regarding reporting the underlying cause of death are expected to be similar across all occupation groups. The large number of records available allow for analysis of more specific occupation and industry groups and causes of death. This study provides baseline (surveillance) data regarding mortality in women miners and compares them to similarly employed women and men, thus reducing the impact of the healthy worker effect, as both groups are likely to suffer loss to employment of ill workers. The PMRs used were considered to be valid when the classification of death in the two populations was similar. This dataset demonstrated this when comparing miners to manufacturing laborers, as seen in the lower level of ill-defined deaths in both occupations. 

Mortality data is limited by poor reporting practices, over-reporting of deaths, and incomplete forms, therefore, it is rated as medium quality [21,41,42]. A previous study by Wilson et al. (2019) validated the use of South African mortality data for occupational mortality studies [43]. Further limitations of the data include that the length of employment or other employment were not collected, although information on the usual or longest-held occupation and industry were available. Misclassification may have been a source of bias due to inaccurate reporting of usual occupation and industry and cause of death. While the degree of misclassification of cause of death varies by disease, fatal chronic disease such as lung cancer is more accurately classified than many other causes of death. Under-reporting of homicides in vital registration data also exists compared to forensic post-mortem data in South Africa [34].

## 5. Conclusions

In this descriptive study, the mortality rates of women in the mining industry were both similar and different to those of men in the mining industry, possibly due to not only biological differences but also social and workplace cultural differences. An excess of deaths from unnatural causes was seen in women miners, which are generally considered to be largely preventable and occur in younger people, resulting in a substantial loss of potential life and a lower age of death in women miners. Thus, these deaths in women miners need to be investigated to describe risk factors in order to develop controls and prevention efforts.

## Figures and Tables

**Table 1:** Demographics at death in the years 2013–2015 for men and women aged 15+, women miners, manufacturing laborers and male miners, manufacturing laborers.

	All Women *n* = 594,114	All Men *n* = 650,857	All Women Miners *n* = 763	Male Miners *n* = 6020	Manufacturing Women Laborers *n* = 541	Manufacturing Male Laborers *n* = 783
**Age at death** Median (years)	60	53	44	55	53	52
**Education** (percentage)						
None	20.1	13.2	10.5	11.3	5.4	10.3
Primary school	33.0	34.9	17.1	39.7	33.3	35.7
High school	43.6	47.9	66.3	46.5	45.9	52.7
Tertiary	3.3	4.0	6.2	2.5	3.7	1.3
**Smoking** (percentage)	14.1	46.8	11.1	47.3	17.3	50.3
**Marital status** (percentage)						
Never married	50.1	53.7	53.6	35.0	53.7	51.4
Married	27.0	36.9	26.7	53.7	21.4	38.1
Divorced	20.4	6.6	16.9	8.1	21.4	7.9
Widowed	2.5	2.7	2.8	3.2	2.6	2.6

**Table 2:** Proportional mortality ratios (PMRs) of external causes of death in miners, manufacturing laborers, and unemployed people.

External Cause of Death	Women Miners PMR (95% CI)	Women Manufacturing Laborers PMR (95% CI)	Unemployed Women	Male Miners PMR (95% CI)	Male Manufacturing Laborers PMR (95% CI)	Unemployed Men PMR (95% CI)
Total external causes (V01-Y34)	**183** (136–246)	**66** (45–97.5)	102 (93.5–110)	**80** (76.2–84)	**76** (64–92)	**152** (147–158)
Car occupant transport accident (V49)	**467** (151–1447)	106 (15–752)	**50** (40–62)	**165** (124–220)	33 (5–231)	**38** (32–45)
Vehicle accidents of unspecified vehicle type (V89)	**243** (127–467)	54 (17–167)	**82** (76–88)	109 (94.8–124)	59 (33–104)	**32** (30–35)
Discharge from unspecified firearm (W34)	**464** (221–974)	0	105 (94–117)	**47** (38–58)	85 (50–144)	**168** (162–174)
Accidental hanging or strangulation (W76)	97 (14–685)	60 (9–425)	**238** (220–258)	**58** (46–73)	91(49–170)	**219** (211–227)
Unspecified exposure to smoke and fire (X09)	93 (13–663)	60 (9–426)	**217** (200–235)	**38** (26–56)	154 (69–343)	**196** (183–209)
Exposure to unspecified factor (X59)	**218** (138–347)	64 (32–129)	**90** (86–95)	**90** (82–99)	**68** (48–98)	**122** (119–125)
Contact with sharp object (Y28)	215 (30.3–1529)	139 (20–983)	**115** (97–137)	**62.8** (41–95)	30 (4–215)	**200** (186–215)
Contact with blunt object (Y29)	**2220** (833–5915)	360 (51–2556)	**1180** (108–1286)	86 (71–103)	72 (37–138)	**139** (133–146)

**Bold**—Significantly increased or decreased.

**Table 3:** A regression sensitivity analysis of the association of industry and deaths due to external causes adjusted for age and education.

Industry	Men	Women
Private Households	Ref	Ref
Not economically active	1.19 0.000	1.44 0.000
Unemployed people	1.39 0.000	1.41 0.000
Unspecified activities	1.04 0.335	1.06 0.106
Other activities	1.40 0.000	1.29 0.000
Growing of crops	1.33 0.000	1.77 0.000
Mining and quarrying	1.36 0.000	2.20 0.000
Manufacture	1.13 0.024	1.23 0.040
Production of electricity	1.37 0.000	2.33 0.000
Building	1.23 0.000	2.39 0.000
Other retail trade	1.28 0.000	1.47 0.000
Repair of motor vehicles	1.15 0.230	-
Other land transport	1.51 0.000	1.86 0.000
Business activities	1.18 0.014	1.64 0.000
Educational services	1.14 0.042	1.48 0.000
Other service activities	1.42 0.000	1.69 0.000
**Age groups**		
15–19	40.97 0.000	14.58 0.000
20–24	53.40 0.000	7.68 0.000
25–29	26.00 0.000	4.33 0.000
30–34	13.15 0.000	3.27 0.000
35–39	8.21 0.000	2.73 0.000
40–44	6.05 0.000	2.65 0.000
45–49	5.10 0.000	2.54 0.000
50–54	4.00 0.000	2.46 0.000
55–59	3.08 0.000	1.92 0.000
60–64	2.39 0.000	1.54 0.000
65–69	1.66 0.000	1.37 0.000
70–74	1.36 0.000	1.18 0.001
75–79	1.15 0.009	1.12 0.016
80+	Ref	Ref
**Education**		
None	Ref	Ref
Primary school	1.25 0.000	1.10 0.001
Secondary school	1.72 0.000	1.42 0.000
Tertiary education	2.42 0.000	2.41 0.000
